# When to take action in food-borne disease outbreaks?

**DOI:** 10.2807/1560-7917.ES.2025.30.41.2500228

**Published:** 2025-10-16

**Authors:** Dirk Werber

**Affiliations:** 1 Institute for Infectious Disease Epidemiology, Austrian Agency for Health and Food Safety, Vienna, Austria

**Keywords:** food-borne infections, infection control, outbreaks, evidence-based medicine, public health policy

## Abstract

Identifying and controlling food-borne disease outbreaks (FBDO) remain important public health objectives. There is plenty of guidance on how to detect potential FBDO, e.g. epidemiologically or microbiologically. Likewise, the conduct of an outbreak investigation has long been codified in steps and adapted for FBDO. However, what is less clear is when to act, mainly when to implement appropriate control measures. This is particularly challenging when the causative agent has not yet been detected in the suspected food vehicle(s). The decision on when to act is complex and depends, besides the available evidence, also on other factors, such as the dynamic of the outbreak or the disease severity. No guidance exists for this decision. Notably, an FBDO investigation provides circumstantial evidence on the culprit, and its careful assessment entails an inherently subjective element. There is a need across sectors and countries in Europe for harmonising the view on what constitutes sufficient evidence for furthering investigations and timely taking action, depending on the outbreak situation. A common understanding and possibly a harmonised legislation are the basis for streamlining discussions and decisions during FBDO, thereby preventing the delay of implementing necessary control measures.

## Introduction

Food-borne disease outbreaks (FBDO), which are predominantly caused by infectious agents or their toxins, come in all shapes and sizes. Their investigation has a long history of success in identifying contaminated food vehicles and stopping the outbreak by implementing appropriate control measures, and in doing so preventing further cases. Through source investigations they also contribute to our understanding of the underlying causes, thereby helping to improve food safety. Parenthetically, FBDO may enable evaluating different surveillance attributes and sometimes, even new causative agents are identified (e.g. [1]). Thus, investigating and controlling FBDO remain important public health and food safety objectives.

Potential FBDO come to the attention of public health in various ways, e.g. by alert clinicians or laboratory personnel, citizen reporting, epidemic intelligence tools, or they could be signalled by the indicator-based surveillance of food- and waterborne diseases. Statistical algorithms for detecting aberrations in surveillance data that may indicate outbreaks are well established [2]. Importantly, whole genome sequencing methods have become an indispensable tool for identifying geographically dispersed FBDO, which are typically caused by a commercial food vehicle and have the potential of affecting many people. Validated protocols exist to facilitate comparison of genomes of patient and food isolates, at least for the pathogens most frequently causing such outbreaks (e.g. non-typhoidal *Salmonella* spp., *Listeria monocytogenes* [3]).

The epidemiological investigation conducted in outbreak situations is similar to those in other contexts [4]. The components of this systematic approach have been codified in steps [4] and taught in field epidemiology and related programmes around the world. These steps have also been adapted for specific settings in FBDO [5-7].

What is less clearly defined is when to act in FBDO, i.e. when to further food safety investigations or implement appropriate control measures, such as a food recall or withdrawal, public warning, consumer advice or temporary closure of a production facility. This decision is challenging as it needs to balance the responsibility to timely protect the public with the need to protect the credibility and reputation of the food producer or food producing industry (and of public health and food safety authorities). The basis for this decision is the evidence implicating a food vehicle or source.

In this viewpoint, I advocate to foster a more common understanding on when and how to act in FBDO across the European Union (EU), particularly to consider all types of evidence for decision-making, and to map the relevant legislation in the Member States (MS) to evaluate the need for harmonisation.

## Evidence in food-borne disease outbreaks

It is helpful to consider what types of evidence can link the consumption of a particular food to a case. In its manual for reporting FBDO, the European Food Safety Authority (EFSA) has laid down useful definitions for this [8]. Accordingly, evidence can be categorised by its nature into being epidemiological, microbiological or the result of product tracing investigations (Table 1). Environmental evidence, such as from inspecting food premises, is omitted from this discussion as it does not provide strong hints on the suspected vehicle. It is, however, useful for identifying the source and cause of the contamination.

**Table 1:** Types of evidence in food-borne disease outbreak investigations that can implicate food vehicle(s)^a^

Type of evidence	Description
Epidemiological^b^	Broadly, hints generated from evaluating food history of cases or from analytical epidemiological comparisons using exposure information from cases
Microbiological	Detection of a causative agent along the food chain (incl. the suspected vehicle) or its environment combined with detection of the agent in human cases
Product-tracing investigation	Process of following the movement of a food product and its constituents through the stages of production, processing and distribution

^a^ Adapted from EFSA’s Manual for reporting on food-borne outbreaks [8].

^b^ Often generated from only a subset of cases, particularly in large outbreaks.

There is a widely held belief that microbiological evidence is superior to other types of evidence in FBDO. Admittedly, identifying an agent in a sample of an unopened food item may very strongly indicate a causal relationship, particularly if the agent is genetically close to those isolated from patients (what constitutes a sufficiently high relatedness among isolates is a subject on its own, see e.g. [9]). Important, however, is that relationships inferred by the phylogenetic relatedness of agents from patients and food or its environment are considered by themselves ’hypotheses‘ [9] and need to be interpreted within the given epidemiological context [10]. Thus, microbiological evidence is neither sufficient on its own to furnish indisputable proof, nor is it a prerequisite for outbreak intervention. Furthermore, microbiological evidence is not easy to obtain. Sampling and testing food samples, particularly in geographically dispersed FBDO, is challenging and often time consuming for at least three reasons: Firstly, microbial contamination is often heterogenous. Usually only few batches of a product, sometimes only one (although possibly comprising a large volume [11]), are contaminated and even within a batch, only a fraction of packing units may contain the causative agent. Secondly, microbial contamination is often at low levels. Furthermore, the pathogen can become internalised through roots, leaves, stems, and flowers into fresh produce items [12]. Combined with the sometimes-challenging food matrix, e.g. through the presence of environmental stressors [13] or PCR inhibitors [14], this may hamper identification of the causative agent (or its genomic content) in food and is sometimes possible only in specialised laboratories. Thirdly, the different routes of investigation to identify the suspected food vehicle are often not conducted in parallel. In geographically dispersed outbreaks, an epidemiological investigation is launched by public health authorities after a cluster of genetically closely related microorganisms from patient specimens has been identified. The aim of this investigation is to identify a suspect vehicle. Food safety authorities rely on this information to tailor product-tracing investigations and microbiological testing of food samples. With the help of the Rapid Alert System for Food and Feed (RASFF), food safety authorities can rapidly exchange information allowing for timely product-tracing investigations across the EU. Nonetheless, targeted sampling and microbiological testing of a suspected food vehicle can often be conducted only after epidemiological investigations and sometimes even only after product-tracing investigations narrowed down the list of suspected food vehicles, producers or batches (non-targeted microbiological investigations, such as comparing the genome of the outbreak strain with isolates from food in available databases, should commence in parallel).

When considering the degree of confidence with which a suspected food vehicle can be linked with outbreak cases, a few general points are worth noting about evidence generated by the investigation of FBDO, as they have implications for both the assessment and the management of FBDO. These points are summarised in Table 2, and some may be illustrated by the investigation of a large outbreak of enteroaggregative Shiga toxin-producing *Escherichia coli* (STEC) O104:H4 in Germany, causing almost 3,500 diarrhoeal cases and ca 850 cases of haemolytic-uraemic syndrome [15]. An early case-control study found an association with the consumption of tomatoes, cucumbers and leafy green salad [16]. When shortly thereafter STEC — not yet characterised by typing methods — was found in samples from cucumbers from Spain, the state of Hamburg informed the public and reported the findings to all EU countries via the RASFF [17]. This was premature given that neither epidemiological nor microbiological evidence convincingly implicated cucumbers as the suspected vehicle of infection (in defence, Hamburg’s population faced a high risk for severe illness at the time). Approximately 2 weeks later, a matched case-control study and a recipe-based restaurant cohort study strongly implicated sprouts as the vehicle. Concurrently conducted product-tracing investigations pointed unequivocally to a single German producer [15]. These results led to a public warning, consumer advice as well as the temporary closure of the production [15]. This investigation exemplifies that the type of evidence is not correlated with its strength (in this case, microbiological evidence was initially weak and missing at the time of the effective intervention), that assessing the strength of evidence must be based on all available evidence and that not all types of evidence must be present to act. Furthermore, it is a case in point that the strength of evidence typically increases with length of the investigation, also illustrating the intricacy of the decision when to act (see also below).

**Table 2:** Important aspects about evidence in food-borne disease outbreaks and their implications

Point	Aspect	Implication
1	The type of evidence is not necessarily correlated with its strength [8].	Focus should be on how convincingly each type of evidence implicates the food vehicle(s) (alongside with other evidence, see Point 4).
2	All types of evidence in outbreaks are circumstantial.	The term ‘evidence‘ in outbreaks should be interpreted as ’hint‘ or ’sign‘, not proof.
3	The strength of evidence^a^ can be viewed as measured on a continuous scale^b^. Theoretically it ranges from absolute certainty to the complete lack thereof.	Assessing the strength of evidence relies on inferences drawn from indirect evidence; it inherently entails a subjective element.
4	The assessment of the strength of evidence should be based on all available types of evidence [8].	As this is a key element, a common understanding is warranted to streamline this process.
5	Not all types of evidence must be present to convincingly implicate a food vehicle.	It may put the public at unacceptable risk to wait until comprehensive evidence is provided (e.g. until microbiological evidence is present).
6	There is a correlation between length of the investigation and strength of evidence.	Since control measures must be implemented rapidly to prevent further cases, there is a trade-off between length of investigation and strength of evidence provided.

EFSA: European Food Safety Authority; FBDO: foodborne disease outbreak.

^a^ In FBDO, strength of evidence reflects the degree of belief of a causal relationship between the suspected food vehicle and outbreak illness.

^b^ For simplicity and practicability, strength of evidence is typically dichotomised into weak and strong, e.g. in EFSA’s FBDO outbreak reporting system, or in guidance documents (e.g. [22]).

## Decision on when to act is multifactorial

All evidence provided by the investigation of outbreaks is circumstantial. In FBDO, it relies on inference to causally connect the suspected food vehicle(s) with illness. Consequently, assessing the evidence entails inherently a subjective component, i.e. it requires judgement. Of note, exemplary guidance from EFSA on what constitutes strong evidence is specifically formulated to support countries in the process of annually reporting FBDO [8]. Although a helpful starting point, this does not equate to guidance for the decision when to act. This decision is multifactorial and not depending solely on the strength of evidence implicating a particular food vehicle. The dynamic of the outbreak, the total number of cases, the severity of associated illnesses and sometimes the economic impact are also influencing factors. No guidance exists thus far for this decision. Plausibly, rapidly rising case numbers and a severe form of disease should lower the threshold to intervene (Figure), i.e. the strength of evidence threshold necessary for implementing control measures should consider the public health risk. In addition, although less often openly debated, a factual consideration in the decision process might be the expected consequence of the intervention — for the incriminated food producer/food producing industry, public health and food safety authorities and their superordinate political offices. As all aforementioned factors are subject to change during an FBDO, the decision on when and how to implement control measures needs to be frequently revisited.

**Figure:**
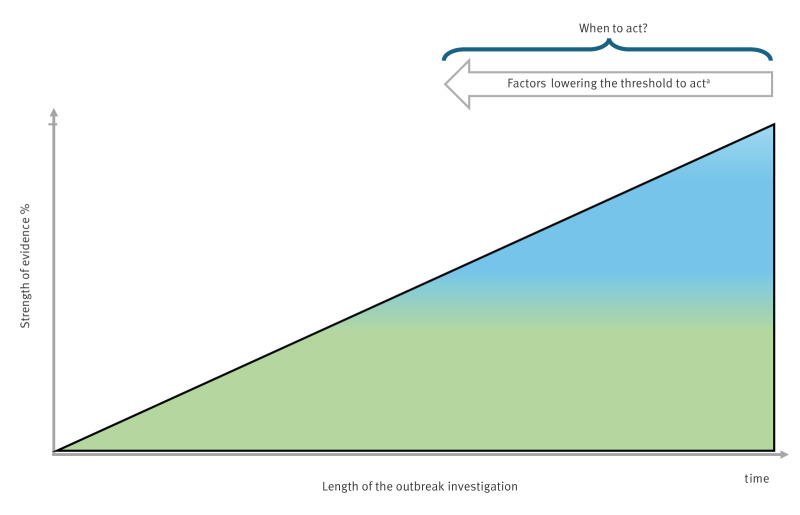
When to further food safety investigations or implement control measures in food-borne disease outbreaks

^a^ For example, rising case numbers, high disease severity, large political or economic impact.

A comprehensive FBDO investigation requires cross-sectoral collaboration, ideally from the outset of the investigation. However, depending on the respective legislation in the country, food safety authorities may themselves require a certain level of evidence from public health authorities before they start conducting in-depth investigations, such as visiting the premises of a food producer and taking samples for microbiological testing. Clearly, the strength of evidence required for these investigations must be lower compared with that for control measures. It is important to properly distinguish between these two situations, i.e. (i) when to further investigations to gather more evidence and (ii) when to implement control measures, based on the existing evidence. For example, in a recent multinational *Salmonella* Umbilo outbreak [18], the epidemiological evidence may not have unequivocally implicated a single food vehicle. Nearly all interviewed cases reported to have eaten leafy green salads in the inquired exposure period, but only 59% reported consumption of rocket salad. Nonetheless, evidence from case interviews was sufficient to initiate product tracing investigations and, commendably, concurrent microbiological testing. The combination of case interviews and trace-back investigations pointed to a single producer, and the causative agent was found in samples from rocket salad (and later baby spinach) from that producer, which led to product recalls and other control measures [18]. This underlines that the threshold for thorough investigations from food safety authorities should be low.

## Common understanding and possibly harmonising the legislation within the EU necessary

It is important to note that each outbreak is unique with respect to dynamic and severity as well as the available evidence. Thus, the decision on when and how to act must be made on a case-by-case basis. However, the assessment when the available evidence implicates a food vehicle with sufficient certainty to implement control measures varies currently between countries and likely even within countries, particularly when the causative agent has not been identified in a food vehicle. A more common understanding, across sectors and countries, is necessary to reduce both overly cautious considerations and sometimes even controversial discussions during FBDO to minimise the delay of implementing necessary control measures. This might be fostered by commonly reviewing hypothetical or past outbreak scenarios or tested by conducting simulation or risk assessment exercises. For example, the *S.* Umbilo outbreak could serve as an exemplary case study, in which one could evaluate if case investigations alone convincingly implicated the food vehicle or, if not, what was missing. In a similar vein, one could ask whether the combination of epidemiological evidence and product-tracing investigations implicated the producer convincingly enough to implement control measures (without the presence of microbiological evidence). Consensually developed guidance, similar to an evidence-to-decision framework [19] and possibly based on such evaluations, would serve both investigation teams and risk managers. It should provide criteria for assessing the strength of evidence, set out what can be realistically achieved and when, and what this entails for the decision process. In cross-border FBDO, countries affected through importation of the suspected vehicle rely greatly on actions taken by the country exporting it. A more consensual view, i.e. greater “inter-investigator-agreement”, combined with transparency in the evidence assessment would lend itself to heightened trust between countries and sectors.

Harmonisation may also be required within the scope of jurisdiction. The Zoonoses Directive 2003/99/EC requires EU MS to epidemiologically investigate FBDO but does not regulate control measures [20]. Consequently, EU countries likely have their own regulations, which are possibly influenced by historical FBDO. For example, a complex binational listeriosis outbreak [21] led to a revision of the respective food safety legislation in Austria. Since then, epidemiological evidence is taken into consideration when implementing control measures. To establish the feasibility and basis for legal harmonisation, a detailed mapping of the current relevant legislation in the EU countries may be worthwhile.

## Conclusion

Food safety is a collaborative effort within the EU and even beyond. International trade of food and supply chains of their ingredients imply risk of cross-border outbreaks. Outbreak investigation is an integral part of food safety, and an established public health tool to identify contaminated food vehicles and to implement appropriate control measures in a timely manner. Its full potential relies on streamlined actions during FBDO, which in turn would benefit from a consensual guidance on when to act and possibly a harmonised legislation — both of which should acknowledge the value of all types of evidence for decision-making.

## Data Availability

All data are presented in the manuscript.
